# The Relationship of Partial Pressure of Carbon Dioxide (PaCO_2_) with Disease Severity Indicators Such as BODE and GOLD in Hospitalized COPD Patients

**DOI:** 10.1155/2022/4205079

**Published:** 2022-03-16

**Authors:** Xiaodiao Zhang, Xiaqi Miao, Keke Ding, Jianing Wang, Binbin Hu, Xueting Hu, Jiamin Shen, Chunyan Liu, Yage Xu, Xiuxiu Zhao, Lulu Bao, Wei Chen, Beibei Zhang, Yiben Huang

**Affiliations:** ^1^Department of Respiratory Medicine, The Third Affiliated Hospital of Wenzhou Medical University, Wenzhou, Zhejiang, China; ^2^School of the First Clinical Medical Sciences, Wenzhou Medical University, Wenzhou, Zhejiang, China; ^3^Department of Gastroenterology, The Third Affiliated Hospital of Wenzhou Medical University, Wenzhou, Zhejiang, China

## Abstract

**Purpose:**

This study aimed to investigate the relationship of partial pressure of carbon dioxide (PaCO_2_) with BODE and GOLD in stable COPD subjects and to explore the predictive value of PaCO_2_ for severe COPD (BODE index score ≥5 or GOLD index score ≥3). *Patients and Methods*. In total, 80 participants with COPD and free from other conditions affecting PaCO_2_ were recruited. Arterial blood gases, BODE, GOLD, SGRQ, lung function, and other data were collected. The BODE index was calculated, and patients were divided into two groups according to the BODE index and PaCO_2_ median, respectively. We used Pearson's correlation test and the receiver operating characteristic curves to evaluate the utility of PaCO_2_. Besides, the univariate and multivariate logistic regression analyses were conducted to verify whether PaCO_2_ was an independent factor associated with BODE grades.

**Results:**

COPD subjects with BODE ≥5 and GOLD ≥3 had significantly higher levels of PaCO_2_ (*p* = 0.004, *p* = 0.001, respectively). In the high PaCO_2_ group, patients underwent poorer outcomes than the low PaCO_2_ group. PaCO_2_ was negatively correlated with forced expiratory volume in 1 second in percent of the predicted value (FEV_1_%) (*r* = −0.612, *p* < 0.001). The performance of PaCO_2_ levels in predicting BODE ≥5 and GOLD ≥3 was 0.748 and 0.755, respectively. The logistic regression analyses proved that PaCO_2_ was associated with BODE ≥5 in COPD patients (odds ratio = 1.160, 95% CI: 1.025–1.313, *p* = 0.019).

**Conclusions:**

A higher level of PaCO_2_ was associated with a higher index for BODE or GOLD in COPD and had the predictive value for severe COPD.

## 1. Introduction

Chronic obstructive pulmonary disease (COPD), characterized by persistent respiratory symptoms, airflow limitation, and recurrent exacerbations [1, 2], is currently one of the leading causes of morbidity and mortality worldwide in the adult population [3]. Murphy et al. reported that persistent hypercapnia after an exacerbation was associated with excess mortality and early rehospitalization [2]. In the study of Dreher et al., a quarter of patients with COPD GOLD III and IV had chronic hypercapnic respiratory failure (CHRF) [4]. The previous study proved the benefits of domiciliary noninvasive ventilation (NIV) in COPD patients with CHRF in terms of COPD Assessment Test (CAT), arterial blood gases (including PaCO_2_), the number of acute exacerbations, and the BODE index of the first 6 months [5]. Home noninvasive ventilation combined with home oxygen therapy prolonged the time to readmission or death among individuals with persistent hypercapnia in acute exacerbation of COPD (AECOPD) [2]. Moreover, a clinical trial reported that the technique veno-venous extracorporeal CO_2_ removal (vv-ECCO_2_R) had the potential to rapidly correct respiratory acidosis and could decrease elevated mean pulmonary artery pressure values in severe COPD significantly by reducing high PaCO_2_ [6]. Almagro et al. stated that partial arterial carbon dioxide tension (PaCO_2_) at discharge was one of the significant predictors of mortality [7]. Additionally, Quintana et al. showed that elevated PaCO_2_ and decreased pH upon emergency department were predictors of ICU or IRCU admission [8]. Multidimensional indexes, such as the body mass index, airflow obstruction, dyspnea, and exercise capacity (BODE index), characterize the severity of COPD in a complex way (based on body mass index (BMI), forced expiratory volume in 1 second (FEV_1_), modified Medical Research Council (mMRC) dyspnea scores, and 6-minute walking distance (6MWD)) [9, 10]. The Global Initiative for Chronic Obstructive Lung Disease (GOLD) stages define the varying severity of COPD [11]. Worse disease severity of COPD can be defined by BODE ≥5 or GOLD ≥3 [9, 11–13]. COPD with hypercapnia was related to severely impaired lung function, and the negative correlation between PaCO_2_ and discharge ambulation distance may reflect the severity of airway obstruction [14]. However, no research has explored the association between PaCO_2_ and severity of patients in stable COPD directly.

Taking these facts into account, the aims of this study were to investigate the relationship of PaCO_2_ with BODE and GOLD in stable COPD subjects and to explore the predictive value of PaCO_2_ for severe COPD (BODE index score ≥5 or GOLD index score ≥3) [9, 11–13].

## 2. Materials and Methods

### 2.1. Study Population

In this cross-sectional research, 80 stable COPD patients were enrolled from the Department of Respiratory Medicine, the Third Affiliated Hospital of Wenzhou Medical University. The recruitment started in February 2018 and ended in February 2019. Inclusion criteria were as follows: (1) age more than 40 years and (2) diagnosis of COPD as defined in the Global Initiative for Chronic Obstructive Lung Disease guidelines; exclusion criteria were as follows: (1) bronchiectasis; (2) asthma; (3) post-tuberculosis sequelae; (4) malignant tumor; (5) chronic heart failure; (6) hepatic and renal insufficiency; (7) clinically significant chest wall deformity; and (8) neuromuscular weakness.

The study protocol was approved by the Ethics Committee of the Third Affiliated Hospital of Wenzhou Medical University, and the registration number of the Ethics Committee was YJ20170015. The study was conducted in accordance with the Declaration of Helsinki. Written informed consent was obtained from all study subjects.

### 2.2. Data Collection

Data such as age, gender, duration of disease, smoking status, and body mass index (BMI) were recorded upon hospital admission. We collected blood samples on 24 hours of admission to analyze blood routine parameters and blood biochemistry. Freshly drawn arterial blood was used for the measurement of pH, partial pressure of carbon dioxide, and oxygen in arterial blood (PaCO_2_ and PaO_2_). Spirometry tests were taken to measure FEV_1_, forced vital capacity (FVC), FEV_1_/FVC, and FEV_1_ in percent of the predicted value (FEV_1_%), which were significant indicators of baseline severity of COPD. Spirometry was conducted to determine the GOLD grades of airflow limitation, which was performed after the administration of an adequate dose of a short-acting inhaled bronchodilator to minimize variability [15]. GOLD 1-2 (FEV_1_ ≥ 50% predicted) indicate low risk, while GOLD 3-4 (FEV_1_ < 50% predicted) indicate high risk. Moreover, composite markers of disease including GOLD (Global Initiative for Chronic Obstructive Lung Disease), BODE (BMI, airway obstruction, dyspnea, and exercise capacity ), mMRC (modified Medical Research Council ), and SGRQ (St. George's Respiratory Questionnaire) were evaluated for every participant. Six-minute walk distance (6MWD) tests were conducted according to the ATS guidelines [16]. It measures the distance that a patient can quickly walk on a 100 ft hallway in a period of 6 minutes, which reflects the exercise capacity for daily physical activities [16].

### 2.3. Statistical Analysis

All statistical analysis was performed using SPSS 25.0 (IBM Analytics). Normally distributed variables were presented as mean ± SD, while variables with skewed distribution were expressed as median (25th–75th percentile). We conducted the independent-samples *t*-test and the Mann–Whitney *U*-test to compare the differences in clinical characteristics between the low BODE group and the high BODE group, and the low PaCO_2_ group and the high PaCO_2_ group. Categorical variables were expressed as counts and percentages, and the chi-squared (*χ*^2^) test or Fisher's exact test was performed for the intergroup comparison. The correlations between PaCO_2_ and numerical variables were estimated using Pearson's or Spearman's correlation coefficients. Pearson's correlation analysis was used to evaluate the correlation between PaCO_2_ and FEV_1_%. The predictive accuracy of PaCO_2_ for higher GOLD and BODE index was determined by calculating the area under ROC curves (AUC). To identify the risk factors associated with COPD severity (higher GOLD and BODE index), we performed univariate analyses. Additionally, the multivariate logistic regression models were carried out to explain the contribution of PaCO_2_ in severe COPD with related confounders adjusted. All effects were considered significant at *p* < 0.05.

## 3. Results

### 3.1. Baseline Characteristics of the Study Participants with Different COPD Severities

**Figure 1:**
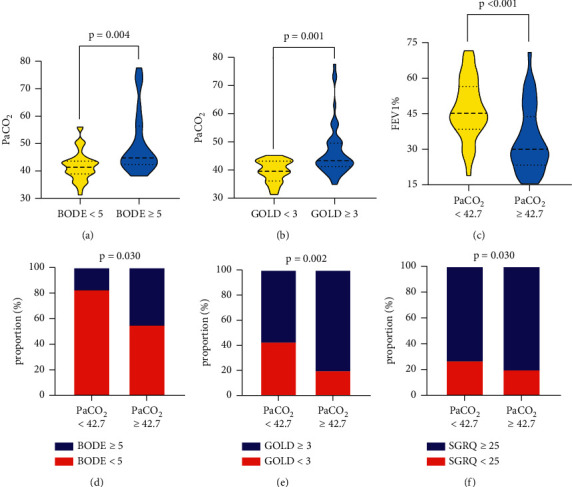
Comparisons of PaCO_2_ according to the BODE and GOLD index, FEV_1_%, and the distribution of BODE, GOLD, and SGRQ index according to the PaCO_2_ median in COPD patients. (a) PaCO_2_ levels of COPD patients according to BODE, *p* = 0.004. (b) PaCO_2_ levels of COPD patients according to GOLD, *p* = 0.001. (c) FEV_1_% of COPD patients according to PaCO_2_ median, *p* < 0.001. (d) The proportion of COPD patients with high BODE scores according to PaCO_2_ median, *p* = 0.030. (e) The proportion of COPD patients with high GOLD scores according to PaCO_2_ median, *p* = 0.002. (f) The proportion of COPD patients with high SGRQ scores according to PaCO_2_ median, *p* = 0.030.

We categorized 80 subjects into two groups according to the BODE index (BODE <5, *n* = 50; BODE ≥5, *n* = 23, 7 patients without BODE index). As shown in Table 1, there were no statistically significant differences in age, sex, duration of disease, proportions of current smokers, pH on day 7 of admission, and PaO_2_ between two groups. Statistical differences were found when it comes to BMI, pH, and FEV_1_%. In addition, the indicator PaCO_2_ of the high BODE group was remarkably higher than the low BODE group, which is described in Figure 1(a) (41.77 ± 5.22 vs. 49.75 ± 11.55, *p* = 0.004). Moreover, the high GOLD group had a drastically higher level of PaCO_2_ compared with the low GOLD group (39.69 ± 3.89 vs. 46.47 ± 9.08, *p* = 0.001, Figure 1(b)).

For the purpose of investigating the relationship between PaCO_2_ and disease severity in COPD patients further, subjects were divided into two groups according to the median of PaCO_2_ (PaCO_2_ < 42.7, *n* = 39; PaCO_2_ ≥ 42.7, *n* = 41). Demographic variables such as age, gender, duration of disease, and proportions of current smokers were insignificantly different between the two groups, as well as the blood routine parameter WBC. There were also no statistical significances in terms of BMI, albumin, mMRC, and PaO_2_. Patients with higher levels of PaCO_2_ had lower levels of pH, pH on day 7 of admission, and all indicators of pulmonary function.  Figure 1(c) shows that subjects with a higher level of PaCO_2_ had a lower level of FEV_1_%, which implies poorer pulmonary function (*p* < 0.001). Besides, GOLD, BODE, and SGRQ were significantly higher in the high PaCO_2_ group, which are described in Table 2.

### 3.2. Correlation of PaCO_2_ with COPD Outcomes

**Figure 2:**
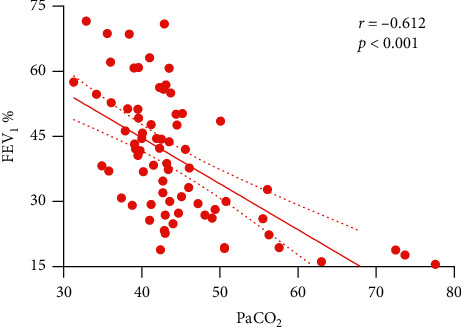
Correlation of the PaCO_2_ with FEV_1_%, *r* = −0.612, *p* < 0.001.

To explore the correlation between PaCO_2_ and scales of COPD outcomes, we used Pearson's or Spearman's correlation analysis.  Table 3 manifested the results that age, BMI, pH, and pH on day 7 of admission were negatively related to PaCO_2_. As the PaCO_2_ increased, the indicators of respiratory function such as FEV_1_, FVC, FEV_1_/FVC, and FEV_1_% dramatically descended.  Figure 2 vividly illustrates the decreasing tendency of FEV_1_% with the PaCO_2_ elevated (*r* = −0.612, *p* < 0.001). Conversely, the scores of GOLD, BODE, and SGRQ grew up markedly with the increased PaCO_2_. No other statistical correlations were found between the rest of the parameters and PaCO_2_.

**Figure 3:**
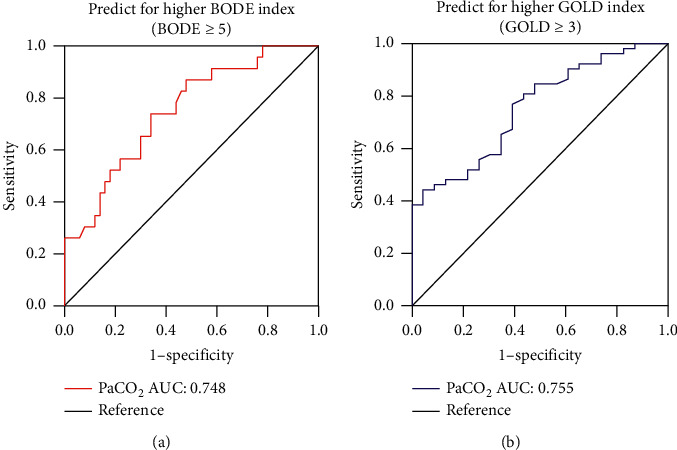
ROC curves of the PaCO_2_ for higher BODE index (BODE ≥5) and higher GOLD index (GOLD ≥3) of COPD patients. (a) The area under ROC curve: 0.748; 95% CI: 0.630–0.866; *p* = 0.001. (b) The area under ROC curve: 0.755; 95% CI: 0.643–0.867; *p* < 0.001.

On the basis of the study results above, we observed the intimate link of PaCO_2_ and GOLD or BODE, so we plotted the ROC curve analysis to evaluate the utility of PaCO_2_ for predicting poorer pulmonary function (GOLD ≥3) and higher disease severity (BODE ≥5) of COPD. The results are shown in Figure 3 that at a cut-off value of 0.399,the sensitivity and specificity of PaCO_2_ in predicting BODE ≥5 were 73.9% and 66.0% , respectively, with an AUC of 0.748 (95% CI: 0.630–0.866, *p* = 0.001). At a cut-off value of 0.399, the sensitivity and specificity of PaCO_2_ for GOLD ≥3 were 44.2% sensitivity and 95.7% specificity, respectively, with an AUC of 0.755 (95% CI: 0.643–0.867, *p* < 0.001). It elucidated that PaCO_2_ had high predictive value for severe COPD (GOLD ≥3 and BODE ≥5).

### 3.3. Increased PaCO_2_ Level Is Related to Higher COPD Severity

In terms of the low PaCO_2_ group, 17.14% suffered from severe COPD (BODE ≥5), while the proportion rose up to 44.74% in the high PaCO_2_ group (*p* = 0.030, Figure 1(d)). Additionally, participants with GOLD ≥3 took up a proportion of 80.00% in the high PaCO_2_ group in contrast with 57.14% in the low PaCO_2_ group (*p* = 0.002, Figure 1(e)). Moreover, in comparison with the low PaCO_2_ group, the high PaCO_2_ group comprised a larger proportion of patients with SGRQ ≥ 25, which represents poorer life quality (*p* = 0.030, Figure 1(f)).

Afterwards, we used univariate logistic regression analyses to determine the independent risk factors for COPD severity according to BODE ≥5. Consequently, BMI (*p* = 0.001), pH (*p* = 0.002), FEV_1_/FVC (*p* = 0.001), FEV_1_% (*p* < 0.001), GOLD ≥3 (*p* = 0.015), mMRC ≥2 (*p* < 0.001), and PaCO_2_ (*p* = 0.003) were observed to have significant relevance with BODE ≥5 in Table 4. Furthermore, the multivariate logistic regression analyses were performed with potential confounders controlled. In Model 1, nothing was adjusted (odds ratio (OR) = 1.146, 95% CI: 1.047–1.253, *p* = 0.003). In Model 2, adjustment for age, sex, smoking status, and duration of disease slightly improved the magnitude of the OR (odds ratio (OR) = 1.160, 95% CI: 1.049–1.284, *p* = 0.004). Even when adjusted for age, sex, smoking status, duration of disease, and FEV_1_/FVC (Model 3), the relationship between PaCO_2_ and BODE ≥5 remained still significant (odds ratio (OR) = 1.160, 95% CI: 1.025–1.313, *p* = 0.019). As can been seen from Table 5, we could draw a conclusion that PaCO_2_ is an independent risk factor for BODE ≥5.

## 4. Discussion

To our knowledge, this study is the first to demonstrate the association of PaCO_2_ with BODE and GOLD index and indicators of pulmonary function in stable COPD subjects. In this study, we found a higher level of PaCO_2_ in COPD patients with BODE ≥5 than BODE <5. Besides, lower pH, higher level of BODE index, and higher level of GOLD index could be observed in patients with higher level of PaCO_2_. Furthermore, our data suggested that elevated PaCO_2_ was associated with other outcome indicators of COPD such as pulmonary indexes, especially lower levels of FEV_1_ and FEV_1_% and higher SGRQ index (SGRQ ≥25), which imply poorer pulmonary function and health-related quality of life in stable COPD, respectively [13, 17]. Univariate and multivariate logistic regression analyses illustrated that increased PaCO_2_ was an independent marker of BODE ≥5 and associated with reduced outcomes in stable COPD. We conducted the ROC curves to estimate the predictive value of PaCO_2_ for severe COPD (defined by BODE ≥5 and GOLD ≥3). The results showed the high accuracy of PaCO_2_ for predicting severe COPD, which may provide some clues to clinic.

Severe COPD patients with GOLD III and IV will develop CHRF [18], which provokes more often exacerbations, shown to be a poor prognostic factor for survival [5, 14, 19]. Ventilation-perfusion inequality always causes an increased arterial PaCO_2_ or hypercapnia in COPD patients [20]. Murphy et al. observed that hypercapnia decreases the secretion of IL-6 and tumor necrosis factor in the lungs and impairs lung neutrophil function in an animal model of lung infection [2]. Hypercapnic patients had lessened ambulation distance and worse function capacity, while the weaker respiratory muscles may be to blame [14].

A previous study showed that prescription of long-term oxygen therapy (LTOT) in patients with COPD and reversible hypercapnia slowed down the natural decline in exercise performance and improved exertional dyspnea [21]. Short-term NIV has become an accepted management approach for patients with CHRF [2]. Moreover, noninvasive positive pressure ventilation in patients with stable hypercapnic and high inspiratory pressures targeted to reduce partial pressure of CO_2_ in arterial blood to some extent can improve survival [22]. Clinical trials have reported the effects of NIV in terms of blood gases and pulmonary function status including FVC, FEV_1_, and quality of life in COPD [2]. The improvement of FVC reflects an amelioration of lung hyperinflation [2]. Our findings were consistent with the literature, which implicated the association of PaCO_2_ and poor outcomes of COPD. Zikyri et al. [5] reported that domiciliary NIV reduced the levels of PaCO_2_ of stable COPD patients with CHRF with a significant improvement in the BODE index. NIV can not only alleviate the airway wall edema to improve the lung function but also relieve fatiguing respiratory muscles by reducing degree of hyperinflation to provide patients a better exercise capacity [2]. Multiple mechanisms contain the improvement of sleep hypoventilation and better ventilation-perfusion (V/Q) matching could attribute to the amelioration of exacerbation number in COPD subjects, especially with hypoxemia during sleep [5]. Moreover, COPD patients with hypercapnia, despite severe ventilatory impairment and weak respiratory muscles, tolerate exercise well and benefit significantly from intensive inpatient pulmonary rehabilitation [14]. Furthermore, vv-ECCO_2_R has been shown to correct severe respiratory acidosis rapidly and decrease elevated systolic pulmonary artery pressure [6]. Besides, it was found previously that there was a strong correlation between pulmonary artery dilatation and severe COPD exacerbation [6]. This clinical trial Karagiannidis et al. [6] conducted could expound the close connection of PaCO_2_ and severity of COPD more directly.

However, there are still several limitations in our study. First, this study is a cross-sectional survey limited to retrospective cohort; thus, further research of PaCO_2_ associated with longitudinal outcomes needs to be conducted to verify the causality. Second, the sample size is relatively small. Third, the inherent mechanisms of the impact of PaCO_2_ on COPD remain to be clarified.

In conclusion, as a simple, reproducible, widely available, and inexpensive predictive tool, PaCO_2_ has a homogeneous and strong effect on severity of stable COPD. Patients with higher levels of PaCO_2_ were associated with poorer outcomes in terms of BODE or GOLD index. Moreover, PaCO_2_ has predictive value for severe COPD (BODE ≥5 or GOLD ≥3). Our preliminary findings seem worthy of attention because PaCO_2_ as a biomarker of COPD severity can be obtained from arterial blood gas analysis fast and conveniently in clinic.

## Figures and Tables

**Table 1:** Baseline characteristics of COPD patients according to disease severity (BODE < 5 or BODE ≥ 5).

	BODE < 5 (*n* = 50)	BODE ≥ 5 (*n* = 23)	*p*
Age (years)	71.26 ± 7.75	70.26 ± 7.72	0.610
Sex (male, *n*%)	41(82.00%)	19 (82.61%)	1.000
Duration of disease (years)	6 (3–12)	11(6–12)	0.051
Smoking (*n*%)	40 (80.00%)	18 (36.00%)	1.000
BMI (kg/m^2^)	22.03 ± 3.51	18.74 ± 3.19	<0.001
pH	7.42 ± 0.03	7.39 ± 0.04	<0.001
pH on day 7 of admission	7.41 ± 0.03	7.39 ± 0.04	0.172
FEV_1_%	44.94 ± 13.73	28.86 ± 11.36	<0.001
PaCO2 (mmHg)	41.77 ± 5.22	49.75 ± 11.55	0.004
PaO2 (mmHg)	74.64 ± 10.62	72.89 ± 17.89	0.665

Data are presented as mean ± SD unless indicated otherwise. COPD, chronic obstructive pulmonary disease; BMI, body mass index; FEV_1_%, forced expiratory volume in 1 second in percent of the predicted value; PaCO2, partial pressure of carbon dioxide in arterial blood; PaO2, partial pressure of oxygen in arterial blood; SD, standard deviation.

**Table 2:** Demographic and laboratory characteristics of COPD patients according to PaCO_2_ median.

	PaCO_2_ < 42.7 (*n* = 39)	PaCO_2_ ≥ 42.7 (*n* = 41)	*p*
Age (years)	72.87 ± 8.10	70.24 ± 7.17	0.128
Sex (male, *n*%)	32 (82.05%)	33 (80.49%)	0.858
Duration of disease (years)	8 (3–12)	11.5 (5.75–21)	0.116
Smoking (*n*%)	29 (74.36%)	32 (78.05%)	0.854
BMI (kg/m^2^)	21.52 ± 3.76	20.48 ± 3.62	0.218
pH	7.42 ± 0.03	7.39 ± 0.04	<0.001
pH on day 7 of admission	7.41 ± 0.04	7.39 ± 0.03	0.035
WBC (×10 ^∗^ 9/L)	8.15 ± 4.04	7.18 ± 2.67	0.209
Albumin (g/L)	34.74 ± 3.66	36.29 ± 2.96	0.049
FEV_1_ (L)	1.12 ± 0.40	0.79 ± 0.29	<0.001
FVC (L)	2.05 ± 0.62	1.63 ± 0.43	0.001
FEV_1_/FVC	54.66 ± 9.41	47.95 ± 9.01	0.003
FEV_1_%	46.96 ± 12.78	34.09 ± 13.85	<0.001
GOLD	2.69 ± 0.68	3.23 ± 0.77	0.002
BODE	3.34 ± 2.07	4.58 ± 2.63	0.030
mMRC	2 (1-2)	1 (1-2)	0.211
SGRQ	34.27 ± 15.42	43.30 ± 20.01	0.030
PaO_2_ (mmHg)	75.80 ± 9.70	70.80 ± 16.49	0.101

Data are presented as mean ± SD unless indicated otherwise. COPD, chronic obstructive pulmonary disease; BMI, body mass index; WBCs, white blood cells; FEV_1_, forced expiratory volume in 1 second; FVC, forced vital capacity; FEV_1_%, FEV_1_ in percent of the predicted value; GOLD, Global Initiative for Chronic Obstructive Lung Disease; BODE, BMI, airway obstruction, dyspnea, and exercise capacity; mMRC, modified Medical Research Council dyspnea score; SGRQ, St. George's Respiratory Questionnaire; PaO_2_, partial pressure of oxygen in arterial blood; PaCO_2_, partial pressure of carbon dioxide in arterial blood; SD, standard deviation.

**Table 3:** Correlations between lung function, severity of disease, other indicators, and PaCO_2_.

Variables	PaCO_2_	
	*r*	*p*
Age (years)	−0.254	0.023
Duration of disease (years)	0.165	0.145
BMI (kg/m^2^)	−0.239	0.034
pH	−0.639	<0.001
pH on day 7 of admission	−0.586	<0.001
WBC (×10 ^∗^ 9/L)	−0.001	0.996
Albumin (g/L)	0.109	0.358
FEV_1_ (L)	−0.523	<0.001
FVC (L)	−0.486	<0.001
FEV_1_/FVC	−0.377	0.001
FEV_1_%	−0.612	<0.001
GOLD	0.558	<0.001
BODE	0.505	<0.001
mMRC	0.177	0.122
SGRQ	0.299	0.008
PaO_2_ (mmHg)	0.169	0.133

COPD, chronic obstructive pulmonary disease; BMI, body mass index; WBCs, white blood cells; FEV_1_, forced expiratory volume in 1 second; FVC, forced vital capacity; FEV_1_%, FEV_1_ in percent of the predicted value; GOLD, Global Initiative for Chronic Obstructive Lung Disease; BODE, BMI, airway obstruction, dyspnea, and exercise capacity; mMRC, modified Medical Research Council dyspnea score; SGRQ, St. George's Respiratory Questionnaire; PaO_2_, partial pressure of oxygen in arterial blood; PaCO_2_, partial pressure of carbon dioxide in arterial blood.

**Table 4:** Univariate logistic regression analyses of factors for BODE ≥ 5.

	OR	95% CI	*p*
Age (years)	0.983	0.922–1.049	0.605
Sex (male)	0.959	0.262–3.510	0.950
Duration of disease (years)	1.050	0.973–1.133	0.213
Smoking	0.900	0.269–3.015	0.864
BMI	0.734	0.607–0.887	0.001
pH^∗^	0.745	0.619–0.897	0.002
WBC	0.859	0.703–1.051	0.140
Albumin	0.886	0.735–1.068	0.205
FEV_1_/FVC	0.884	0.821–0.952	0.001
FEV_1_%	0.902	0.854–0.953	<0.001
GOLD			
<3	1.000		
≥3	0.145	0.031–0.693	0.015
mMRC			
<2	1.000		
≥2	0.034	0.008–0.145	<0.001
SGRQ			
<25	1.000		
≥25	0.222	0.046–1.070	0.061
PaCO_2_	1.146	1.047–1.253	0.003
PaO_2_	0.990	0.952–1.029	0.596

COPD, chronic obstructive pulmonary disease; BMI, body mass index; WBCs, white blood cells; FEV_1_, forced expiratory volume in 1 second; FVC, forced vital capacity; FEV_1_%, FEV_1_ in percent of the predicted value; GOLD, Global Initiative for Chronic Obstructive Lung Disease; BODE, BMI, airway obstruction, dyspnea, and exercise capacity; mMRC, modified Medical Research Council dyspnea score; SGRQ, St. George's Respiratory Questionnaire; PaO_2_, partial pressure of oxygen in arterial blood; PaCO_2_, partial pressure of carbon dioxide in arterial blood; pH∗, the data were present according to pH∗100.

**Table 5:** Adjusted odds ratio (95% confidence interval) for BODE ≥ 5.

Variables	OR	95% CI	*p*
Model 1	1.146	1.047–1.253	0.003
Model 2	1.160	1.049–1.284	0.004
Model 3	1.160	1.025–1.313	0.019

Model 1 is univariate analysis. Model 2 is adjusted by age, sex, smoking status, and duration of disease. Model 3 is adjusted by age, sex, smoking status, duration of disease, and FEV_1_/FVC.

## Data Availability

The datasets generated and/or analyzed during this study are not publicly available due to individual privacy but are available from the corresponding author on reasonable request.

## Abbreviations

COPD: Chronic obstructive pulmonary disease
PaCO_2_: Partial pressure of carbon dioxide in arterial blood
PaO_2_: Partial pressure of oxygen in arterial blood
FEV_1_%: Forced expiratory volume in 1 second in percent of the predicted value
FEV_1_: Forced expiratory volume in 1 second
FVC: Forced vital capacity
BODE: Body mass index, airway obstruction, dyspnea, and exercise capacity
GOLD: Global Initiative for Chronic Obstructive Lung Disease
SGRQ: St. George's Respiratory Questionnaire
mMRC: Modified Medical Research Council dyspnea score
CHRF: Chronic hypercapnic respiratory failure
NIV: Noninvasive ventilation
AECOPD: Acute exacerbation of chronic obstructive pulmonary disease
LTOT: Long-term oxygen therapy
vv-ECCO_2_R: Veno-venous extracorporeal CO_2_ removal
BMI: Body mass index
6MWD: 6-Minute walking distance
ROC: Receiver operating characteristics
AUC: The area under the curve.
